# Magnesium and Nitrogen Co-Doped Mesoporous Carbon with Enhanced Microporosity for CO_2_ Adsorption

**DOI:** 10.3390/nano8050275

**Published:** 2018-04-25

**Authors:** Jingting Lu, Chengli Jiao, Zeeshan Majeed, Heqing Jiang

**Affiliations:** 1 Qingdao Key Laboratory of Functional Membrane Material and Membrane Technology, Qingdao Institute of Bioenergy and Bioprocess Technology, Chinese Academy of Sciences, No. 189 Songling Road, Qingdao 266101, China; lujt@qibebt.ac.cn (J.L.); jiaocl@qibebt.ac.cn (C.J.); zeeshan@qibebt.ac.cn (Z.M.); 2 University of Chinese Academy of Sciences, Beijing 100049, China

**Keywords:** mesoporous carbon, Mg doping, steam activation, basic site, microporosity, CO_2_ adsorption

## Abstract

Mesoporous carbons (MC) have attracted a tremendous amount of interest due to their efficient molecular transport properties. However, the limited number of active sites and low microporosity generally impede their use for practical applications. Herein, we have fabricated Mg and N co-doped mesoporous carbon (Mg-NMC) with high microporosity via one-pot synthetic route followed by further steam activation. In comparison with the parent N-doped mesoporous carbon, Mg-NMC shows partially ordered mesostructure and improved CO_2_ adsorption capacity attributed to the introduction of basic site after Mg doping. Upon further steam activation, the microporosity is enhanced to 37.3%, while the CO_2_ adsorption capacity is also increased by 70.4% at 273 K and 1.0 bar.

## 1. Introduction

Mesoporous carbons (pore size 2–50 nm) have attracted a great deal of attention in diverse areas including gas adsorption, catalysis and energy storage [1], owing to their unique physicochemical properties such as good chemical inertness and mesostructured channels. Compared to microporous carbon materials, the channels of mesoporous carbons facilitate fast mass transfer [2]. However, mesoporous carbon materials generally possess a limited number of active sites, which hinders their use for practical applications. Incorporation of heteroatoms such as B, N, P, S and F into carbon framework has been widely explored to increase the number of active sites of carbon materials [3,4,5,6]. In particular, the incorporation of N atoms not only increases number of basic sites but also improves surface polarity, which would significantly enhance the catalytic and adsorption properties of carbon materials [7]. For instance, Zhao et al. introduced N atoms into carbon framework to obtain N-doped mesoporous carbon with a high N content and a CO_2_ adsorption capacity of 2.8–3.2 mmol g^−1^ at 298 K and 1.0 bar [8]. However, most mesoporous carbons have a low N content or mesopore structure with low thermal stability [9], mainly due to the decomposition of N-containing precursors during self-assembly process and N-containing carbon framework during the carbonization at high temperature [8].

Recently, different metal species have been dispersed into N-containing carbon frameworks for improving adsorption and catalytic properties [10,11]. Some of these metal species may stabilize the preformed nitrogen sites [12]. In addition, previous literatures have suggested that MgO nanoparticles embedded into mesoporous carbon can provide a large number of basic O^2−^ sites which are beneficial for CO_2_ adsorption, and restrict further shrinkage of carbon framework [13,14]. On the other hand, Mg^2+^ can accelerate the polycondensation process, and improve the cure rate of phenolic resin [15].

In addition to basic active sites, microporosity is also one of the most important factors governing CO_2_ adsorption properties. It has been demonstrated that micropores with pore size less than 1 nm have strong adsorption affinity for CO_2_ molecules. Based on previous literature, microporosity of carbon materials can be easily enhanced by physical as well as chemical activation [16]. Currently, KOH, CO_2_, NH_3_ and steam have been widely explored as common activation agents for the development of microporosity [17]. In contrast to other activation agents, steam has attracted intensive attention on account of being readily available, cost effective and environmentally benign. Han et al. reported that graphene aerogel with steam activation exhibited a higher CO_2_ adsorption capacity (2.5 mmol g^−1^ at 273 K and 1.0 bar) than sample without steam activation. In addition, the Langmuir specific surface area of graphene aerogel without steam activation was 820 m^2^ g^−1^, which was increased to 1690 m^2^ g^−1^ after steam treatment [18]. The enhanced surface area and CO_2_ adsorption performance were attributed to high microporosity resulting from steam activation.

The above discussion implies that the introduction of basic sites by doping heteroatoms or metal species coupled with enhanced microporosity by physicochemical activation play an important role in manipulating the adsorption properties of mesoporous carbon materials. However, the work reported so far mainly emphasized on how to increase the adsorption capacity either by tunable surface modification or adjustable microporosity. In this study, we combine these two strategies to enhance the CO_2_ adsorption properties of mesoporous carbon. At first, basic N and Mg species were incorporated into carbon framework using 3-aminophenol and hexamethylentetramine as nitrogen source and Mg(NO_3_)_2_·6H_2_O as magnesium source, resulting in partially ordered mesostructure with two types of basic sites. In the second step, the as-synthesized Mg and N co-doped mesoporous carbon was etched by steam at elevated temperature to generate a large number of micropores in the carbon framework. As expected, after steam activation, the Mg and N co-doped material exhibited a much higher CO_2_ uptake capacity.

## 2. Materials and Methods

### 2.1. Materials

All chemicals were utilized as received without further purification: Pluronic F127 (Mw = 12,600 EO_106_-PO_70_-EO_106_, Sigma-Aldrich, Co., St. Louis, MO, USA), Magnesium nitrate hexahydrate (Sinopharm Chemical Regent Co., Ltd., Shanghai, China), 3-aminophenol (3-AP, Aladdin Industrial Corporation, Shanghai, China) and hexamethylentetramine (HMT, Aladdin Industrial Corporation, Shanghai, China).

### 2.2. Synthesis of N-Containing Mesoporous Carbon and Mg and N Co-Doped Mesoporous Carbon

N-containing mesoporous carbon (NMC) and Mg and N co-doped mesoporous carbon (Mg-NMC) were prepared via a procedure similar to that reported by Wang et al. [19]. In a typical synthesis, 0.654 g of 3-AP, 0.42 g of HMT and 0.534 g of Mg(NO_3_)_2_·6H_2_O were dissolved in 75 mL of deionized water at room temperature. Then, 5 mL of deionized water containing 0.47 g of Pluronic F127 was slowly added into the above solution with stirring. The resulting mixture was further stirred at room temperature for 30 min to obtain a homogeneous solution. After that, it was transferred to a thermostated oil bath to continue stirring at 50 °C for 16 h, followed by stirring at 80 °C for 8 h to ensure complete polymerization. The obtained polymer was collected by filtration, washed with deionized water and dried at 50 °C under vacuum. The dried sample was further calcined at 350 °C for 2 h followed by calcination at 700 °C for 3 h with a heating rate of 1 °C min^−1^ under argon flow.

### 2.3. Steam Activation

Steam activation was performed in a quartz tube furnace, wherein Mg-NMC was heated from room temperature to 700 °C at a ramp rate of 5 °C min^−1^ under a flow of argon. After the furnace temperature reached at 700 °C, the carrier gas was bubbled through water before entering into the tube furnace and the furnace temperature was maintained at 700 °C for 4 h. Finally, the carrier gas was switched back to pure argon in order to prevent uncontrolled activation during the cool down process. The final sample was denoted as Mg-NMC-H_2_O.

### 2.4. Characterization

X-ray photoelectron spectroscopy (XPS) measurements were performed on an ESCALAB 250Xi with Mg Kα (hν = 1253.6 eV) X-ray source (Thermo Fisher Scientific, Waltham, MA, USA). Fourier-transform infrared (FT-IR) spectra were recorded on a Nicolet iN10 IR microscope (Thermo Nicolet Corporation, Madison, WI, USA). Transmission electron microscopy (TEM) analyses were carried out using an H-7650 microscope (Hitachi High-Technologies Corporation, Tokyo, Japan) and a G2 microscope (FEI Co., Hillsboro, OR, USA) operated at an accelerate voltage of 120 kV. Nitrogen adsorption and desorption isotherms and CO_2_ adsorption isotherms were carried out on an Autosorb-iQ analyzer (Quantachrome Instruments, Boynton Beach, FL, USA ) at 77 K and 273 K, respectively. Prior to measurements, all samples were degassed under vacuum at 200 °C for 8 h. Surface areas were calculated using Brunauer-Emmett-Teller (BET) method. Pore size distributions were calculated via the nonlocal density functional theory (NLDFT) algorithm. Microporosity was calculated from the relation X% = V_mico_/V_total_ × 100%. XRD patterns were obtained with a Bruker D8 Advance diffractometer (Bruker AXS GmbH, Karlsruhe, Germany) using Cu Kα radiation source. Scanning electron microscopy (SEM) studies were conducted with Hitachi S-4800 microscopes (Hitachi, Tokyo, Japan). CO_2_ temperature programmed desorption (CO_2_-TPD) experiment was carried out on an AutoChem II 2920 chemisorption analyser (Micromeritics Instrument Corporation, Norcross, GA, USA). Before experiment, the sample was degassed at 700 °C for 2 h under helium flow, and then cooled down to room temperature. The sample was exposed to flowing 10% CO_2_/He for 2 h, and heated from room temperature to 900 °C under helium flow with a heating rate of 10 °C min^−1^.

## 3. Results and Discussion

**Figure 1:**
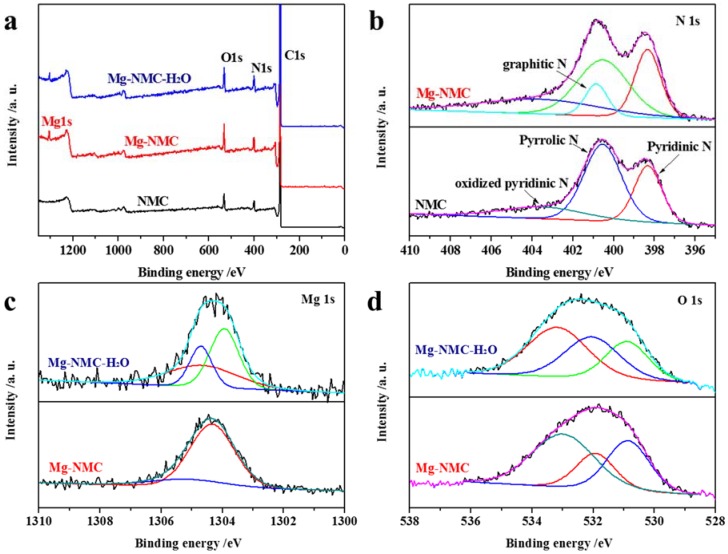
(**a**) XPS survey spectra for NMC, Mg-NMC and Mg-NMC-H_2_O; (**b**) XPS N 1s spectra of NMC and Mg-NMC; (**c**) XPS Mg 1s and (**d**) O 1s spectra of Mg-NMC and Mg-NMC-H_2_O.

NMC was fabricated via soft-template method using 3-AP and HMT as precursors and F127 as surfactant under alkaline conditions. Addition of magnesium salt into the precursors of NMC produced Mg-NMC. The sample Mg-NMC-H_2_O was obtained by further etching of Mg-NMC in water vapor at elevated temperature. XPS analyses were performed to investigate the chemical composition of all the samples. As shown in Figure 1a, the XPS spectra of NMC and Mg-NMC exhibit strong signals of carbon, nitrogen and oxygen elements, verifying the presence of N in the two samples. The N content of NMC reaches up to 10.9 wt %, which is higher than the value reported in previous literature. Additionally, the XPS spectrum of Mg-NMC shows Mg 1s signal, indicating a successful incorporation of Mg within the mesoporous matrix. In order to investigate the types of nitrogen species present in NMC before and after Mg doping, N 1s XPS analysis was performed and the corresponding spectra are presented in Figure 1b. Three peaks are observed in the N 1s curve-fitting spectrum of NMC. The peak at 398.3 eV is assigned to pyridinic N, the strong peak at 400.6 eV is attributed to pyrrolic N, while the small peak at 403.2 eV can be ascribed to oxidized pyridinic N [20,21]. After Mg doping, the N 1s spectrum shows a new peak at 400.8 eV which is attributed to graphitic N [2]. Therefore, it can be reasonably speculated that Mg doping leads to the generation of graphitic N sites in carbon framework. Both pyridinic and graphitic N are considered as basic sites [22,23]. Magnesium and oxygen states of Mg-NMC before and after steam activation were investigated in detail. As shown in Figure 1c, Mg 1s XPS spectrum of Mg-NMC consists of two peaks located at 1304.4 and 1304.7 eV which indicate the presence of MgO and MgCO_3_ [24,25]. This can be explained on the following grounds. Initially, Mg(NO_3_)_2_ fully decomposed to MgO during the calcination of Mg-NMC at 700 °C. MgO probably converted to MgCO_3_ and Mg(OH)_2_ in situ by reaction with CO_2_ and water released from the carbonization of phenolic resin and decomposition of triblock copolymer [26]. Subsequently, these Mg species could decompose either partially or completely back to MgO under high temperature conditions. Apart from MgCO_3_, a small amount of Mg(OH)_2_ might also be present, but it is difficult to observe due to the low peak intensity [27]. After steam activation, three peaks were observed at 1303.9, 1304.7 and 1305 eV respectively in the Mg 1s spectrum of Mg-NMC-H_2_O (Figure 1c, upper). The peak at 1303.9 eV indicates the presence of Mg(OH)_2_, which is probably formed by the reaction between MgO/MgCO_3_ and adsorbed water during the steam activation process [28]. Figure 1d displays the O 1s XPS spectra of Mg-NMC and Mg-NMC-H_2_O. Before steam activation, the O 1s curve-fitting spectrum of Mg-NMC contains three peaks located at 530.9 eV (MgO); 531.9 eV (C-O); and 533.0 eV (MgCO_3_) [29,30,31]. In contrast, after steam activation, central peak shifts from 531.9 to 532.1 eV, which can be ascribed to the combination of C-O and Mg(OH)_2_ [29]. These results suggest that Mg-NMC-H_2_O probably contains MgO, MgCO_3_ and Mg(OH)_2_. However, it is difficult to ascertain these magnesium species by XPS analysis, which can be further confirmed by FT-IR measurement.

**Figure 2:**
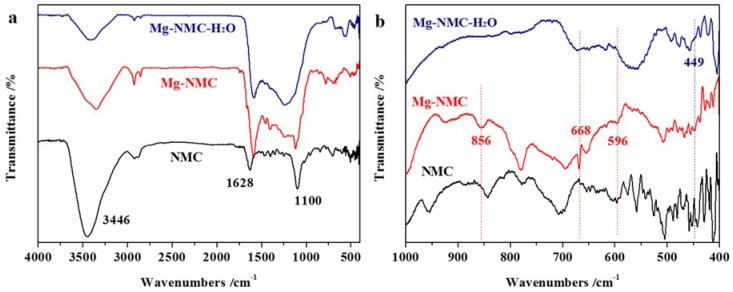
FT-IR spectra (**a**,**b**) of NMC, Mg-NMC and Mg-NMC-H_2_O.

The FT-IR spectra of NMC, Mg-NMC and Mg-NMC-H_2_O are depicted in Figure 2. As shown in Figure 2a, the featured absorption peaks assigned to N-containing carbons are observed for NMC, such as 3446 cm^−1^ (N-H stretching vibration), 1628 cm^−1^ (C=C and C=O stretching vibration) and 1100 cm^−1^ (C-N or C-O stretching vibration) [32,33,34]. These results further confirm the existence of nitrogen species in NMC. After incorporation of Mg into the NMC framework (Figure 2b), two absorption peaks in the finger print region at 668 and 596 cm^−1^ can be assigned to Mg-O stretching and bending vibrations, suggesting the formation of MgO in Mg-NMC [35,36]. In addition, the absorption peak at 856 cm^−1^ is ascribed to the out-of-plane bending vibrations of CO_3_^2−^ [37]. After steam activation, a new observed peak at 449 cm^−1^ can be assigned to Mg-O stretching vibration in Mg(OH)_2_ [38]. All these results demonstrate that MgO and MgCO_3_ co-exist in Mg-NMC, whereas after steam activation Mg(OH)_2_ is generated in the framework of Mg-NMC-H_2_O.

**Figure 3:**
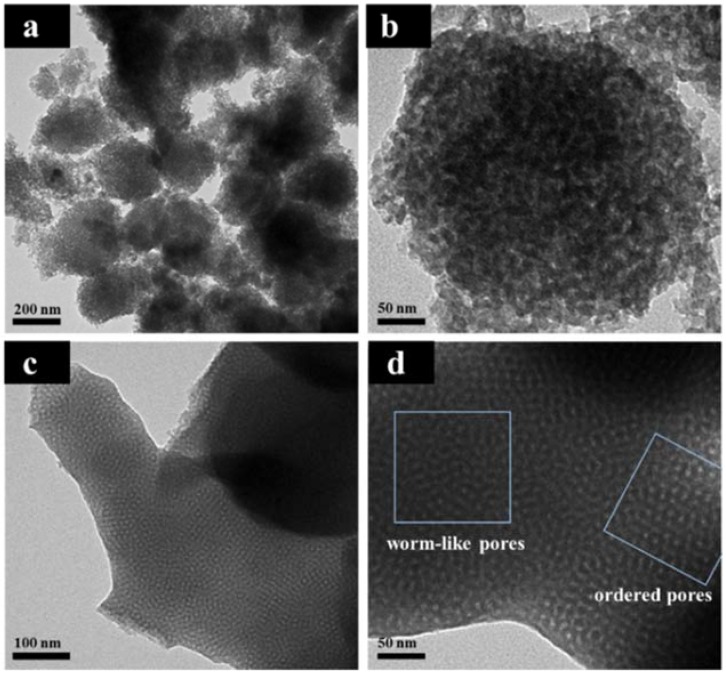
TEM images of NMC (**a**,**b**) and Mg-NMC (**c**,**d**).

In order to further probe the influence of Mg doping on the morphology and mesostructure of NMC, TEM analyses were performed. As shown in Figure 3a and b, the parent NMC exhibits spherical morphology with a disordered mesostructure. However, after Mg doping the sample Mg-NMC shows a combination of ordered and worm-like mesostructure (Figure 3c,d). This enhanced mesostructure ordering is mainly attributed to Mg doping. During the self-assembly process, Mg^2+^ can improve the polymerization of ortho-methylol groups to generate methylene linkages, leaving more para-methylol groups which can easily react with similar groups from other molecules resulting in considerable shortening of cure time of the resin [39]. Previous reports have also suggested that enhancing polymerization [40] or polycondensation [41] rate contributes towards improvement of structural ordering. As seen in Figure 3, the disordered mesostructure is changed to partially ordered mesostructure after Mg doping, indicating the efficacy of Mg^2+^ as a catalyst for the construction of ordered mesostructure. Furthermore, no isolated particles are observed either outside or on the surface of Mg-NMC matrix (Figure 3c,d), which probably refers to a high dispersion of MgO and/or MgCO_3_ within the mesoporous framework without any microphase separation.

**Figure 4:**
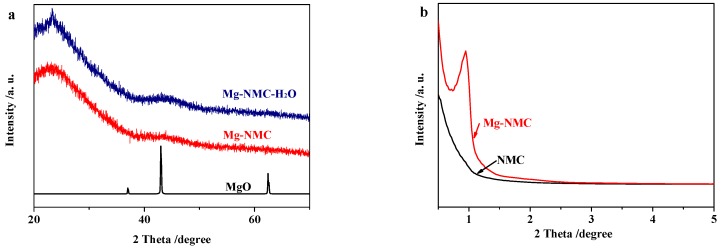
(**a**) Wide-angle XRD patterns of MgO, Mg-NMC and Mg-NMC-H_2_O; (**b**) Small-angle XRD patterns of NMC and Mg-NMC.

The wide-angle XRD pattern of Mg-NMC presents no apparent diffraction peaks of MgO and MgCO_3_ (Figure 4a), suggesting small particle size as well as high dispersion of magnesium species within the Mg-NMC matrix. Small-angle XRD patterns of NMC and Mg-NMC are displayed in Figure 4b. There is no peak in the small-angle XRD pattern of NMC, indicating its disordered mesostructure. In contrast, a strong diffraction peak around 0.94° is observed for Mg-NMC, which implies that ordered mesostructure can be obtained by the addition of Mg(NO_3_)_2_. These results are in good agreement with the TEM results.

**Figure 5:**
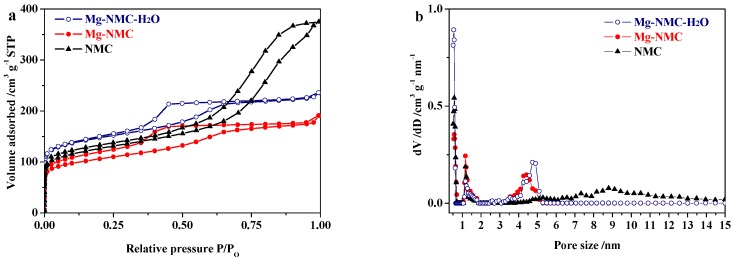
N_2_ sorption isotherms (**a**) and pore size distribution plots (**b**) of NMC (solid triangles), Mg-NMC (solid circles) and Mg-NMC-H_2_O (open circles).

Effect of Mg doping and steam activation on textural features such as microporosity and specific surface area was deeply investigated by N_2_ adsorption measurements. Figure 5 displays the N_2_ sorption isotherms and pore size distribution plots of NMC, Mg-NMC and Mg-NMC-H_2_O. Both NMC and Mg-NMC exhibit typical type IV adsorption curves. However, there are some differences in the shapes of hysteresis loops. The sample NMC displays a typical H_1_-type hysteresis loop and a sharp capillary condensation at high relative pressure range (P/P_o_ > 0.5). In contrast, Mg-NMC exhibits an H_2_-type hysteresis loop and an adsorption saturation platform at a relative pressure of about 0.5. This indicates the improvement of mesostructure ordering for Mg-NMC, which further validates the TEM and small-angle XRD results. Moreover, the pore size of NMC decreases from 9 to 4.5 nm after Mg doping (Figure 5b). A comparison of the textural properties of NMC, Mg-NMC and Mg-NMC-H_2_O is given in Table 1. The specific surface area of NMC reduces from 457 to 385 m^2^ g^−1^. However, the microporosity of NMC increases from 18.3% to 29.8%. These results indicate that Mg doping has a profound influence on the formation of mesostructure during the synthesis process.

After steam activation, Mg-NMC-H_2_O exhibits a much sharper slope at the low relative pressure region (Figure 5a). This phenomenon is due to additional micropores generated by steam etching of Mg and N co-doped carbon framework. During the steam activation process, steam acts as an oxidizing agent for carbon, with a concomitant release of H_2_ and CO which results in the formation of additional micropores. Compared to Mg-NMC, the increased microporosity (from 29.8% to 37.3%) and higher specific surface area (from 385 to 541 m^2^ g^−1^) for Mg-NMC-H_2_O are mainly attributed to these additional micropores. As shown in Figure 5b, the number of micropores with pore size around 0.55 nm significantly increases after steam activation, implying that steam activation can generate more micoropores (below 1 nm). Indeed, it is well known that high microporosity is beneficial for carbon materials to adsorb gas molecules.

**Figure 6:**
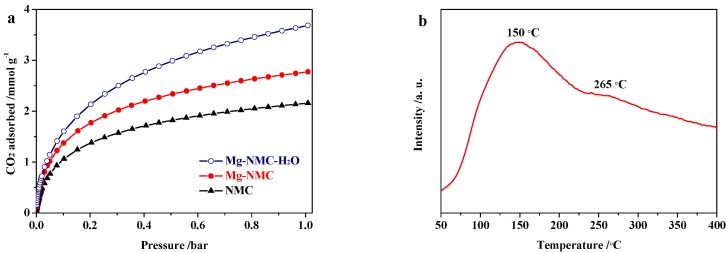
(**a**) CO_2_ adsorption isotherms for NMC, Mg-NMC and Mg-NMC-H_2_O at 273 K; (**b**) CO_2_-TPD profile of Mg-NMC.

The CO_2_ adsorption capacities of NMC, Mg-NMC and Mg-NMC-H_2_O were investigated at 273 K and corresponding adsorption isotherms are depicted in Figure 6a, while Figure 6b displays the CO_2_-TPD profile of Mg-NMC. As can be seen in Figure 6a, before Mg doping, NMC shows an adsorption capacity of 2.16 mmol g^−1^ at 1.0 bar, which increases to 2.77 mmol g^−1^ after incorporation of Mg species into the carbon matrix. Upon steam activation, the adsorption capacity is further enhanced and reaches up to 3.68 mmol g^−1^ at 1.0 bar, which is much higher compared to the inactivated sample. In general, the CO_2_ adsorption capacity of a mesoporous material can be improved by the introduction of basic active sites as well as enhanced microporosity. According to the XPS results previously discussed, Mg doping leads to the generation of basic graphitic N sites in addition to pyridinic and pyrrolic N sites inside the carbon matrix. In addition, the CO_2_-TPD profile of Mg-NMC (Figure 6b) presents two distinct desorption peaks corresponding to two different types of basic sites i.e., N [42] and O^2−^ [43]. In addition, Mg-NMC also possesses higher microporosity (29.8%) compared to NMC (18.3%) as given in Table 1. These results account for the improved CO_2_ uptake capacity of Mg-NMC over NMC. Subsequently, steam activation not only increases the microporosity and specific surface area of Mg-NMC, but also exposes more basic sites to the gas molecules, thereby leading to a further enhancement of CO_2_ uptake capacity. In addition, it is known that suitable micropores with pore size <1 nm have strong adsorption affinity for CO_2_ molecules. For example, Jaroniec et al. have reported that high micropore volume with small pore size (typically less than 0.8 nm) is essential for enhancing CO_2_ adsorption capacity [44]. Likewise in our case, the enhanced adsorption capacity can also be partly attributed to increased microporosity owing to the micropores with pore size around 0.55 nm for Mg-NMC-H_2_O (Figure 5b). As a result, after Mg doping followed by steam activation, the CO_2_ adsorption capacity of NMC increases by 70.4%, implying that basic active sites and microporosity are significantly important for improving the adsorptive performance of mesoporous carbon materials.

## 4. Conclusions

In summary, Mg and N co-doped mesoporous carbon with high microporosity was successfully prepared via one-pot self-assembly method followed by steam activation. In contrast to N-doped mesoporous carbon, Mg and N co-doped mesoporous carbon showed two types of basic sites and partially ordered mesostructure. Upon further steam activation, the microporosity was enhanced to 37.3%, while the specific surface area was increased from 385 to 541 m^2^ g^−1^. Moreover, the Mg and N co-doped mesoporous carbon exhibited a good CO_2_ adsorption capacity of 3.68 mmol g^−1^ at 273 K and 1.0 bar. The present strategy paves the way towards synthesizing heteroatom and metal oxide co-doped mesoporous carbons with high porosity for potential applications in gas adsorption and catalysis.

## Figures and Tables

**Table 1:** Textural properties of NMC, Mg-NMC and Mg-NMC-H_2_O.

Sample	S_BET_/m^2^ g^−1^	V_total_/cm^3^ g^−1^	V_micro_/cm^3^ g^−1^	V_meso_/cm^3^ g^−1^	Microporosity/%
NMC	457	0.58	0.106	0.476	18.3
Mg-NMC	385	0.30	0.088	0.212	29.8
Mg-NMC-H_2_O	541	0.36	0.136	0.224	37.3
